# Periodontal health in an indigenous Sámi population in Northern Norway: a cross-sectional study

**DOI:** 10.1186/s12903-020-01098-3

**Published:** 2020-04-10

**Authors:** Ann-Kristine Sara Bongo, Magritt Brustad, Nils Oscarson, Birgitta Jönsson

**Affiliations:** 1 Department of Community Medicine, Faculty of Health Sciences, UiT the Arctic University of Norway, Tromsø, Norway; 2 The Public Dental Health Service Competence Centre of Northern Norway (TkNN), P.O Box 2406, N-9271 Tromsø, Norway; 3 Sámi University of Applied Science, Kautokeino, Norway; 4 Department of Periodontology, Institute of Odontology, The Sahlgrenska Academy, University of Gothenburg, Gothenburg, Sweden

**Keywords:** Epidemiology, Alveolar bone loss, Periodontitis, Indigenous, Sámi, Oral health

## Abstract

**Background:**

The aim of the study was to describe prevalence, severity and distribution of periodontal disease as well as associated risk factors in an indigenous Sámi population in Northern Norway, and to investigate differences between the indigenous Sámi and the non-Sámi population.

**Methods:**

This cross-sectional study included data from the Dental Health in the North study (*N* = 2078; 18–75 years). Data on Ethnicity, household income, education, smoking habits, dental attendance, and tooth brushing habits were collected by a questionnaire. Periodontal conditions were assessed by clinical examination. A modified version of the new AAP/EFP classification system of periodontal disease was used to estimate the severity of periodontitis. Three stages were used: ‘Non-severe periodontitis’, ‘Stage II’, and stage ‘III/IV’.

**Results:**

Of the total study population 66.5% reported Sámi affiliation. The total prevalence of periodontitis was 49.7%, with 20.1% in Stage III/IV, but no differences between Sámi and non-Sámi. When controlled for sex, age, education, smoking and dental attendance the Sámi had higher probability of having more severe stages of periodontitis; Odds Ratio_Stage II_ (OR) = 1.3; 95% CI: 1.1–1.7; and OR_Stage III/IV_ (OR) = 1.6; 95% CI: 1.1–2.2) compared to non-Sámi. The Sámi had higher prevalence of periodontal pocket depth (PD) ≥ 4 mm (t = 1.77; *p* <  0.001) and PD ≥ 6 mm (t = 1.08; *p* = 0.038) than the non-Sámi.

**Conclusions:**

The prevalence of periodontitis was high in communities in the core area of Sámi settlement in Northern Norway, regardless of ethnicity. People with Sámi ethnicity had more deep periodontal pockets and an increased odds of having severe stages of periodontitis. Future studies should address possible explaining factors behind the potential higher risk of having more severe periodontitis among indigenous people in Sámi settlements.

## Background

The Sámi are the indigenous people living in the region called Sápmi, which today encompasses northern parts of Norway, Sweden and Finland and the Kola Peninsula in Russia [1]. The Sámi people are the minority in Norway and the Norwegian parliament has acknowledged the ethnic group, Sámi, as the only indigenous people in the nation. Due to legal restrictions on the registration of ethnic minority identity, estimating the size of the indigenous Sámi today is not straightforward, though it is currently assumed that Norway has the largest proportion of the total Sámi population.

At present, there are no peer-reviewed studies on periodontal conditions among the Sámi population published in Norway, Sweden, Finland, or Russia. Holst and colleagues [2] published a study in Norwegian of adults (25–60 years old) in Northern Norway back in the 1980’s. They reported that the prevalence of periodontitis was 62%, but only a small part of the study population had severe periodontitis. They found periodontitis to be associated with the region and oral hygiene habits, but not with ethnicity. Studies on indigenous people worldwide have found that indigenous populations generally have poorer oral health than their non-indigenous counterparts [3–5]. The prevalence of periodontitis in indigenous people in Australia [6, 7], Canada [8], New Zealand [9] and USA [10] is reported to be higher than in their non-indigenous counterparts, and the odds of having advanced periodontal disease is also higher [11, 12].

Knowledge about the oral health status of the adult population in Norway in general is quite scanty. For northern Norway, periodontal conditions in an adult population (20–69 years) in a coastal community were described by a study from 1979 [13], and for Troms county by a more recent study of a random sample of adults (20–79 years), in which about half of the study population had periodontitis [14]. Other Norwegian studies have described the periodontal conditions in an age cohort of 35 year olds in Oslo [15], and in a random sample of the elderly population in Norway (> 67 years old) [16]. Studies from Jönköping in Sweden have reported a decrease in the prevalence of periodontitis in adults (20–80 years), from 57% in 1983 to 40% in 2013 [17–19], but the prevalence of severe periodontitis has remained almost the same over the same decades: 16% in 1983 and 11% in 2013 [19]. Also in a Finnish adult population (30–65+ years) [20] the prevalence of deep periodontal pockets has slightly decreased over the last decade, however with difference between genders. In 2011, the prevalence was 21% in men and 14% in women.

The Norwegian government has asked for and allocated resources to research on the oral health of the Sámi population because of the lack of scientific research-based knowledge [21]. In an indigenous context, research on the periodontal conditions in an adult Sámi population is both important and necessary. At present, we know very little about the oral health of the Norwegian indigenous population, notwithstanding that nearly all indigenous studies report that indigenous people worldwide have poorer oral health than their non-indigenous counterparts [4, 5, 8].

The aim of the study was to describe prevalence, severity and distribution of periodontal disease as well as associated risk factors in an indigenous Sámi population in Northern Norway. A further aim was to investigate differences regarding periodontal disease between the indigenous Sámi and the non-Sámi population.

## Methods

### Study population

To describe periodontal conditions in the Sámi population in Northern Norway, data from the “Dental Health in the North” study was used. This study and its methodology is described in detail in Brustad et al. [22]. The study was a cross-sectional study of adults 18–75 years old in Finnmark County in Northern Norway. Data was collected between February 2013 and May 2014. All patients attending public dental care services in five municipalities (Tana, Nesseby, Porsanger, Karasjok and Kautokeino) during the study period were invited to participate in the study regardless of the reason for their appointment at the clinic. Of a total of 2520 persons invited to participate, 285 persons declined (crude response rate at 88.7%) and 157 participants were not included in the final sample. Reasons for non-inclusion were the following: missing questionnaire, missing clinical data or both, unknown target age, missing written consent, or not accounted for and thus given missing unknown status. The final sample consisted of 2078 participants.

The regional committee for medical and health research ethics of the University of Tromsø, Norway, approved the study (2012/1902/REK Nord). All participants provided written informed consent.

### Questionnaire

Population characteristics were collected by self-reported questionnaire. The questionnaire covered information about ethnicity, household income, education, smoking habits, use of dental health care services, and oral hygiene related behaviours. Self-reported ethnicity was based on three questions: 1) Which language do/did you/your parents/grandparents speak at home? 2) What is your/ your parents’ ethnic background, and 3) What ethnicity do you consider yourself to be? The response options were ‘Norwegian’, ‘Sámi’, ‘Kven’ and ‘other’. For a more thorough description of questions included in the questionnaire, including ethnic categorization, see Brustad et al. [22]. Brustad et al. describe the ethnicity as a complex phenomenon, were both the objective factors (parents and grandparents being Sámi) and the subjective feelings of belonging to the Sámi culture have to be taken into account when creating the ethnicity variable [22]. In this study, ethnic affiliation was categorised as ‘Sámi’ and ‘non-Sámi’. The ‘Sámi’ category represented those who answered ‘Sámi’ on at least one of the two questions about language and ethnic background, in addition, reported that they consider themselves as Sámi. All other respondents were categorised as ‘non-Sámi’ even though some of the participants reported that they had Sámi heritage but did not consider themselves as Sámi. The ‘non-Sámi’ group were mainly Norwegians, Kven (without Sámi affiliation, *n* = 99) and Sámi with some affiliation, but without subjective Sámi criteria (*n* = 165).

Education in Norway is mandatory for all children aged 6–16 years. The education system is made up of primary school (6 years), secondary school (4 years), High school (3 years) and Higher education (University level). Participants’ educational level was assessed with one question that elicited responses in number of years, and grouped into three categories: [1] 1–9 years, [2] 10–13 years and [3] ≥14 years. Tooth brushing habits were assessed with one question with four response options from two or more times per day to never. These four options were merged into three categories: [1] less than daily, [2] 1 time/day, and [3] ≥ 2 times/day. Smoking habits were assessed with two questions 1) Do you smoke daily? 2) How many cigarettes do you smoke per day? Age was divided into four age groups: 18–34, 35–49, 50–69 and 65–75 year olds.

### Clinical dental examination

Nine dentists and six dental hygienists with assisting nurses, in six separate dental offices, carried out the clinical examination. Data on a participant’s periodontal condition was collected from a clinical examination including four bitewing radiographs. Periodontal probing depth (PD) at six sites per tooth was measured to the nearest millimetre with a periodontal probe with single millimetre graduations (WHO- probe LM555B). Clinical attachment level (CAL) was not assessed, so the alveolar bone level (ABL) based on the radiographs was used as main criteria classifying prevalence and severity of periodontitis. Third molars and implants were examined but excluded from analysis.

Calibration of the examiners were done as follows: First, the examiners had a workshop regarding the diagnostic criteria and examination procedures. Secondly, all examiners were trained and calibrated towards an experienced periodontist who was the gold standard (NO). This calibration included radiographic examination technique and periodontal pocket probing on one patient each. Third, the examiner (A-KSB) was trained by an experienced periodontist (NO). ABL based on radiographs of randomly chosen participants were measured and an inter-examiner reliability was conducted, κ = 0.97. Examiner (A-KSB) measured the ABL twice, and intra-examiner reliability for the ABL measurements was conducted, κ =0.95.

The clinical dental examination procedure and the post clinical measurements of bone level, including validity, is described in details elsewhere [22].

#### Classification of periodontitis

A modified classification system based on the new AAP/EFP^1^classification system of periodontal disease was used to present prevalence of periodontitis [23, 24]. In this study, stages of periodontitis were classified by radiographic bone loss (RBL) and PD. Missing teeth and furcation involvement were not included. Stage I is the borderline between gingivitis and periodontitis and represents the early stages of attachment loss. Because RBL < 15% can be difficult to measure on radiographs without having the exact root length, Stage I was not included as a periodontal case. A patient was classified as a Stage II periodontitis case if the RBL was between 15 and 33% and as Stage III/IV if the RBL was extending to middle or apical third of the root in two or more non-adjacent teeth. The complexity factor (PD) was included and may shift the stage to the higher level; PD 4–5 mm classified to Stage II and PD ≥ 6 mm classified to stage III/IV. Cases with no periodontitis and early stage of periodontitis were classified as ‘Non-severe periodontitis’ (NSP).

### Statistical analysis

Missing data occurred at a low frequency (0.1–5.0%). There were no internal loss in regards to ethnicity. The greatest proportion of loss was for household income followed by education (3.3%).

Differences between the Sámi- and the non-Sámi groups, as well as classification of periodontal disease, were calculated for demographic and socioeconomic status (age, sex, household income and education), smoking habits and tooth brushing frequency. Prevalence of periodontitis was presented as the frequency distribution for AAP/EFP classification method and presented as ‘Non-severe periodontitis’, ‘Stage II’ and Stage ‘III/IV’. Differences in prevalence of stages of periodontitis between Sámi and non-Sámi was stratified by age group, and assessed with z-test and analysed by univariate regression analysis.

Differences in background characteristics between Sámi and non-Sámi group and between classifications of periodontitis were assessed with Pearson χ^2^ test, and differences between groups were assessed with z-test. Age and number of teeth were presented as means and standard deviation (SD).

RBL and PD are presented as means (standard error [SE] and proportions (SE) of affected sites and teeth for the total study population, stratified by age group and ethnicity. Differences between groups were assessed with z-test and t-test.

Multinomial logistic regression was performed to determine the relationship between stages of periodontitis in relation to ethnicity, socio-demographic and behavioural factors. Ethnicity, sex, age, education, smoking and dental service attendance were used as independent variables. The logistic regression model was done in two steps: 1) associations for each variable with the odds of having different stages of periodontitis were studied in a univariate model. 2) Multivariate models were used to study the adjusted associations. The analysis in the univariate and the multinomial regression were done first in the total population, were the ethnicity was one of the confounding variables and secondly in Sámi population and non-Sámi population separately. ‘NSP’ and ‘Stage II’ were used as reference categories. Differences were assessed using Odds Ratio and 95% confidence intervals. In all analyses the significance level was set at 0.05. Data were analysed using the IBM® SPSS® Statistics, version 25.

## Results

Of the total 2078 participants included in the study, 1381 (66.5%) reported Sámi affiliation. The mean number of teeth in the total study population was 25.1 (SD 3.8). There were no differences in mean number of teeth between Sámi and non-Sámi (25.1 vs. 25.2, *p* = 0.173). The mean age for the participants with Sámi affiliation was 46.7 (SD 14.7) years and 48.9 (SD 13.4) years for non-Sámi (*p* <  0.001). The mean age of all participants was 47.5 (SD 14.3) years. There were more participants with Sámi affiliation in the youngest age group compared to the other age groups (Table 1). More participants with non-Sámi affiliation had a higher yearly household income (> 900,001) than the participants with Sámi affiliation. On the other hand, in the Sámi population more participants had longer education (≥14 years). A majority of the population brushed their teeth at least twice a day, but around 10% of the Sámi participants reported brushing their teeth less than once a day.

**Table 1:** Characteristics of study participants stratified by ethnic affiliation

	Total n (%)	Sámi n (%)	Non-Sámi n (%)	***P*** value*	Internal loss n (%)
Participants	2078 (100%)	1381 (66.5)	697 (33.5)		
Age				< 0.001	0 (0)
18–34	419 (20.1)	313 (22.7)^a^	106 (15.2)^b^		
35–49	687 (33.1)	435 (31.5)^a^	252 (36.2)^b^		
50–64	709 (34.1)	468 (33.9)	241 (34.6)		
65–75	263 (12.7)	165 (11.9)	98 (14.1)		
Sex				0.155	0 (0)
Men	894 (43.0)	579 (41.9)	315 (45.2)		
Women	1184 (57.0)	802 (58.1)	382 (54.8)		
Household income				0.035	104 (5.0)
< 300,000	276 (14.0)	184 (13.3)	92 (13.2)		
300,001-600,000	792 (40.1)	547 (39.6) ^a^	245 (35.2)^b^		
600,001-900,000	615 (31.2)	400 (29.0)	215 (30.9)		
> 900,001	291 (14.7)	174 (12.6) ^a^	117 (16.8)^b^		
Education				< 0.001	69 (3.3)
1–9 years	242 (12.0)	178 (13.4) ^a^	64 (9.3)^b^		
10–13 years	781 (38.9)	462 (34.9) ^a^	319 (46.6)^b^		
≥ 14 years	986 (49.1)	684 (51.7)^a^	302 (44.1)^b^		
Smoking				0.198	28 (1.3)
Yes	443 (21.3)	283 (20.5)	160 (23.0)		
No	1607 (77.3)	1079 (78.1)	528 (75.8)		
Toothbrushing frequency				< 0.001	3 (0.1)
< 1 time/day	175 (8.4)	142 (10.3) ^a^	33 (4.7)^b^		
1 time/day	631 (30.4)	462 (33.5) ^a^	169 (24.3)^b^		
≥ 2 times/day	1269 (61.2)	775 (56.1) ^a^	494 (70.9)^b^		
Dental attendance				0.004	20 (1.0)
Yearly	1150 (55.3)	729 (52.8) ^a^	421 (60.4)^b^		
Every other year	721 (34.7)	501 (36.3) ^a^	220 (31.6)^b^		
Seldom	187 (9.0)	136 (9.9)	51 (7.3)		

*P-value for differences between groups using the χ^2^ test. When numbers in columns do not equal n or 100%, there is an internal drop out in background data. Different superscript letters denotes significant differences in periodontitis prevalence between characteristics (row proportions) at the 0.05 level

### Prevalence and distribution of periodontitis

The prevalence of periodontitis (Stage II and III/IV) was 49.7%, with 20.1% in Stage III/IV. The estimated prevalence and distribution of periodontitis by ethnicity, age and gender, as well as socioeconomic status, smoking habits, toothbrushing habits, and frequency of dental visits are presented in Table 2 for the total sample and in Table 3 for the Sámi participants only. There were no significant differences between Sámi and non-Sámi in the distribution of disease related to stages (Table 2). However, in the Sámi group, the prevalence and severity of periodontitis increased with age; in the oldest age group, a vast majority had periodontitis, and of those 36.4% were classified in Stage III/IV. In the youngest age group, 4.2% had periodontitis and 1.6% had Stage III/IV periodontitis. The prevalence of severe periodontitis decreased with increasing education level / years at school both for the total sample, and for the Sami subsample separately (Tables 2 and 3). More men than women had periodontitis Stage III/IV and more participants attending dental services yearly were classified as Stage III/IV compared to those attending every other year or less often.

**Table 2:** Distribution of periodontitis in relation to demographic, socioeconomic and behavioral factors for the total sample, *n* = 2078

	Periodontitis			
	NSP n (%)	Stage II n (%)	Stage III + IV n (%)	***P*** value
Total	1046 (50.3)	615 (29.6)	417 (20.1)	
Ethnicity				0.630
Sámi	693 (50.2)	403 (29.2)	285 (20.6)	
Non-Sámi	353 (50.6)	212 (30.4)	132 (18.9)	
Age				< 0.001
18–34	400 (95.5)^a^	13 (3.1)^a^	6 (1.4)^a^	
35–49	453 (65.9)^b^	152 (22.1)^b^	82 (11.9)^b^	
50–64	142 (20.0)^c^	332 (46.8)^c^	235 (33.1)^c^	
65–75	51 (19.4)^c^	118 (44.9) ^c^	94 (35.7)^c^	
Gender				0.001
Men	418 (46.8)^a^	265 (29.6)	211 (23.6)^a^	
Women	628 (53.0)^b^	350 (29.6)	206 (17.4)^b^	
Household income				0.884
< 300,000	133 (48.2)	91 (33.0)	52 (18.8)	
300,001-600,000	398 (50.3)	231 (29.2)	163 (20.6)	
600,001-900,000	314 (51.1)	182 (29.6)	119 (19.3)	
> 900,000	145 (49.8)	83 (28.5)	63 (21.6)	
Education				< 0.001
1–9 years	74 (30.6)^a^	77 (31.8)^a^	91 (37.6)^a^	
10–13 years	382 (48.9)^b^	245 (31.4)^a^	154 (19.7)^b^	
> 14 years	562 (57.0)^c^	275 (27.9)^a^	149 (15.1)^c^	
Smoking				< 0.001
Yes	159 (35.9)^a^	145 (32.7)	139 (31.4)^a^	
No	875 (54.4)^b^	461 (28.7)	271 (16.9)^b^	
Toothbrushing				0.220
< 1 time a day	99 (56.6)	40 (22.9)	36 (20.6)	
1 time a day	322 (51.0)	180 (28.5)	129 (20.4)	
> 2 times a day	623 (49.1)	394 (31.0)	252 (19.9)	
Frequency of dental visits				< 0.001
Yearly	517 (45.0)^a^	351 (30.5)	282 (24.5)^a^	
Every other year	419 (58.1)^b^	204 (28.3)	98 (13.6)^b^	
Seldom	102 (54.5) ^b^	55 (29.4)	30 (16.0)^b^	

NSP; No severe periodontitis, Stage II and stage III/IV classified according to a modified AAP/EFP classification method. *P-value for differences between groups using the χ^2^ test.

Differences between groups were assessed with z-test. Different superscript letters denotes significant differences in periodontitis prevalence between characteristics (column proportions) at the 0.05 level

**Table 3:** Distribution of periodontitis in relation to demographic, socioeconomic and behavioral factors for participants reporting Sami affiliation, *n* = 1381

	Periodontitis			
	NSP n (%)	Stage II n (%)	Stage III + IV n (%)	***P*** value
Total	693 (50.2)	403 (29.2)	285 (20.6)	
Age				< 0.001
18–34	300 (95.8)^a^	8 (2.6)^a^	5 (1.6)^a^	
35–49	278 (63.9)^b^	101 (23.2)^b^	56 (12.9)^b^	
50–64	81 (17.4)^c^	223 (47.6)^c^	164 (35.0)^c^	
65–75	34 (20.6)^c^	71 (43.0)^c^	60 (36.4)^c^	
Gender				0.011
Men	271 (46.8)^b^	167 (28.8)	141 (24.4)^b^	
Women	422 (52.6)^a^	236 (29.4)	144 (18.0)^a^	
Household income				0.602
< 300,000	90 (48.8)	63 (34.4)	31 (16.8)	
300,001-600,000	267 (48.8)	163 (29.8)	117 (21.4)	
600,001-900,000	205 (51.2)	109 (27.3)	86 (21.5)	
> 900,000	88 (50.6)	47 (27.0)	39 (22.4)	
Education				< 0.001
1–9 years	57 (32.0)^a^	55 (30.9)	66 (37.1)^a^	
10–13 years	223 (48.3)^b^	140 (30.3)	99 (21.4)^b^	
> 14 years	391 (57.1)^c^	192 (28.1)	101 (14.8)^c^	
Smoking				< 0.001
Yes	100 (35.3)^a^	90 (31.8)	93 (32.9)^a^	
No	583 (54.0)^b^	309 (28.7)	187 (17.3)^b^	
Toothbrushing				0.406
< 1 time a day	79 (55.6)	32 (22.6)	31 (21.8)	
1 time a day	235 (50.9)	133 (28.8)	94 (20.3)	
> 2 times a day	378 (48.8)	237 (30.6)	160 (20.6)	
Frequency of dental visits				< 0.001
Yearly	325 (44.5)^a^	222 (30.5)	182 (25.0)^a^	
Every other year	292 (58.3)^b^	134 (26.7)	75 (15.0)^b^	
Seldom	70 (51.5)^c^	44 (32.3)	22 (16.2)^b^	

NSP; No severe periodontitis, Stage II and stage III/IV classified according to a modified AAP/EFP classification method. *P-value for differences between groups using the χ^2^ test

Differences between groups were assessed with z-test. Different superscript letters denotes significant differences in periodontitis prevalence between characteristics (column proportions) at the 0.05 level

There were no significant differences in prevalence of NSP, Stage II and Stage III/IV periodontitis between Sámi and non-Sámi stratified by age group. In Table 4 prevalence of radiographic bone loss (RBL) and periodontal pocket depth (PD) for the Sámi and the non-Sámi group are presented in age groups. The prevalence of RBL and PD increased with age in both ethnic groups. In total (18–75 years), the participants with Sámi affiliation had significantly higher prevalence of PD ≥ 4 mm (t = 1.77; *p* < 0.001) and PD ≥ 6 mm (t = 1.08; p = 0.038) than the participants in non-Sámi group.

**Table 4:** Prevalence and extent of radiographic bone loss and periodontal pocked depth by age group, ethnicity and in total

Periodontal measurements	Age Groups (years)								Total	
	18–35		35–49		50–64		65–75		18–75	
	Sámi	Non-Sámi	Sámi	Non-Sámi	Sámi	Non-Sámi	Sámi	Non-Sámi	Sámi	Non-Sámi
RBL, % (SE)										
Prevalence										
RBL 15–33%	3.8 (1.1)	5.6 (2.3)	**32.6 (2.3)**	25.0 (2.7)*^c^	59.4 (2.3)	53.5 (3.2)	53.9 (3.9)	57.1 (1.9)	37.7 (1.3)	36.4 (1.8)
RBL > 33%	0.3 (0.3)	0.0 (0.0)	3.5 (0.9)	5.6 (1.4)	23.3 (1.9)	21.2 (2.6)	25.5 (2.7)	25.5 (4.4)	12.1 (0.8)	12.9 (1.2)
PD, % (SE)										
Prevalence										
PD ≥ 4 mm	40.6 (2.8)	40.6 (4.8)	63.7 (2.3)	56.4 (3.1)	**71.6 (2.1)**	63.1 (3.1)*^c^	73.3 (3.4)	81.3 (3.9)	**62.3 (1.3)**	59.8 (1.9)*^a^
PD ≥ 6 mm	5.1 (1.2)	5.7 (2.2)	**14.7 (1.7)**	10.3 (1.9)*^b^	**27.8 (2.6)**	19.9 (2.6)*^c^	27.3 (3.4)	27.5 (4.5)	**18.5 (1.0)**	15.4 (1.4)*^c^
PD, mean (SE)										
Proportions of sites/mouth										
PD ≥ 4 mm	3.5 (0.4)	3.8 (0.7)	7.7 (0.5)	6.8 (0.7)	10.5 (0.7)	10.1 (1.0)	**8.8 (0.9)**	11.7 (1.4)*^a^	8.0 (0.3)	8.2 (0.5)
PD ≥ 6 mm	0.2 (0.0)	0.1 (0.1)	0.5 (0.1)	0.4 (0.1)	1.3 (0.2)	1.1 (0.2)	1.2 (0.2)	1.2 (0.3)	0.8 (0.1)	0.7 (0.1)

RBL = radiographic bone loss; PD = periodontal pocket depth. SE = standard error. Bold-face = statistically significant differences between Sámi and non-Sámi in each age group. *^a^ = *p* < 0.05; *^b^ = *p* < 0.01; *^c^ = *p* < 0.001. Statistical analyses were done with independent t-test for each age group

### Periodontitis association to ethnicity, socio-demographic and behavioural factors

In the univariate analysis of the total population (Table 5), the odds of having severe periodontal disease (Stage III/IV) were associated with age, low education level, smoking and attended dental services yearly. Men had higher probability of having periodontitis than women, with the highest odds observed for Stage III/IV (OR = 1.7; 95% CI: 1.2–2.4). When the model was adjusted for all significant variables in the multivariable model, the odds of having stage III/IV of periodontitis was significantly higher among those with Sámi affiliation compared to non-Sámi (adjusted Odds Ratio (aOR) = 1.6; 95% CI: 1.1–2.2).

**Table 5:** Periodontitis stage II and stage III/IV in relation to demographic, socioeconomic and behavioral factors

Background characteristics	NSP vs. Stage II (total ***n*** = 1661)		NSP vs. Stage III/IV (total ***n*** = 1463)	
	Unadjusted OR (95% CI)	Adjusted OR (95% CI) ***n*** = 1633	Unadjusted OR (95% CI)	Adjusted OR (95%CI) ***n*** = 1434
Ethnicity				
Sámi	1.0 (0.8–1.2)	1.3 (0.9–1.7)	1.1 (0.9–1.4)	**1.6 (1.1–2.2)**
Non-Sámi	Ref.	Ref.	Ref.	Ref.
Sex				
Men	1.1 (0.9–1.4)	**1.3 (1.1–1.7)**	**1.5 (1.2–1.9)**	**1.7 (1.2–2.4)**
Women	Ref.	**Ref.**	Ref.	Ref.
Age (years)				
65–75	**6.9 (4.7–10.0)**	**9.7 (6.4–14.9)**	**10.2 (6.7–15.4)**	**12.2 (7.6–19.7)**
50–64	**7.0 (5.3–9.1)**	**8.4 (6.2–11.2)**	**9.1 (6.7–12.5)**	**10.6 (7.3–15.1)**
35–49	Ref.	Ref.	Ref.	Ref.
18–34	**0.1 (0.05–0.2)**	**0.1 (0.06–0.2)**	**0.08 (0.04–0.2)**	**0.1 (0.04–0.2)**
Education (years)				
1–9 years	**2.1 (1.5–3.0)**	0.9 (0.6–1.4)	**4.2 (2.9–6.0)**	**2.0 (1.3–3.1)**
10–13 years	**1.3 (1.1–1.6)**	1.3 (0.9–1.7)	**1.4 (1.1–1.8)**	**1.5 (1.1–2.1)**
> 14 years	Ref.	Ref.	Ref.	Ref.
Smoking				
Yes	**1.7 (1.3–2.2)**	**2.9 (2.1-4.1)**	**2.8 (2.1–3.7)**	**4.4 (3.0–6.4)**
No	Ref.	Ref.	Ref.	Ref.
Dental attendance				
Seldom	1.1 (0.8–1.6)	1.4 (0.9–1.6)	1.3 (0.8–2.0)	1.6 (0.8–2.9)
Every other year	Ref.	Ref.	Ref.	Ref.
Yearly	**1.4 (1.1–1.7)**	1.2 (0.9–1.5)	**2.3 (1.8–3.0)**	**2.0 (1.4–2.9)**

NSP = non-severe periodontitis. Ref. = Reference group. Adjusted for all variables in the model. In the adjusted model participants with missing values are excluded. Bold = significant differences compared to reference group

In the multivariable analyses (Table 6) for the subpopulations (Sámi and non-Sámi, *n* = 1972), the strongest associations of having periodontitis were age, education and smoking. The adjusted odds of having periodontal disease increased with increasing age in both groups. The odds of having severe periodontitis was also consistently associated with smoking. In the Sámi group likelihood of having periodontitis, stage III/IV, was higher among men compared to women and in adults with less than a high school education (< 14 years).

**Table 6:** Ethnicity-stratified Odds ratio for Periodontitis stage II and stage III/IV by demographic, socioeconomic and behavioral factors

Background characteristics	NSP vs. Stage II (total n = 1661)		NSP vs. Stage III/IV (total n = 1463)	
	Adjusted OR (95% CI)		Adjusted OR (95% CI)	
	Sámi	Non-Sámi	Sámi	Non-Sámi
	***n*** = 1078	***n*** = 555	***n*** = 956	***n*** = 478
Sex				
Men	**1.4 (1.1–2.0)**	1.2 (0.8–1.9)	**2.1 (1.4–3.0)**	1.2 (0.7–2.1)
Women	Ref.	Ref.	Ref.	Ref.
Age (years)				
65–75	**8.5 (5.0–14.4)**	**11.9 (6.0–23.7)**	**10.9 (6.0–20.0)**	**15.7 (7.3–34.8)**
50–64	**8.8 (6.1–12.9)**	**7.5 (4.5–12.2)**	**11.6 (7.3–18.3)**	**9.1 (4.9–17.0)**
35–49	Ref.	Ref.	Ref.	Ref.
18–34	**0.08 (0.4–0.2)**	**0.1 (0.06–0.4)**	**0.1 (0.04–0.3)**	**0.07 (0.01–0.5)**
Education (years)				
1–9 years	0.8 (0.5–1.4)	1.1 (0.5–2.6)	**2.0 (1.2–3.5)**	**2.5 (1.1–5.7)**
10–13 years	1.3 (0.9–1.8)	1.2 (0.8–1.9)	**1.8 (1.2–2.9)**	1.1 (0.6–1.9)
> 14 years	Ref.	Ref.	Ref.	Ref.
Smoking				
Yes	**2.3 (1.5–3.6)**	**4.0 (2.3–7.0)**	**4.7 (2.9–7.6)**	**4.5 (2.3–8.6)**
No	Ref.	Ref.	Ref.	Ref.
Dental attendance				
Seldom	**1.9 (1.1–1.9**)	0.9 (0.3–2.5)	1.4 (0.6–2.8)	2.9 (0.9–9.5)
Every other year	Ref.	Ref.	Ref.	Ref.
Yearly	1.4 (0.9–3.0**)**	0.8 (0.5–1.3)	**2.1 (1.4–3.2)**	**1.9 (1.1–3.6)**

NSP = non-severe periodontitis. Ref. = Reference group. Adjusted for all variables in the model. In the adjusted model participants with missing values are excluded. Bold = significant differences compared to reference group

## Discussion

This study showed that there were no difference in prevalence of periodontitis between Sámi and non-Sámi. However, the Sámi had more deep periodontal pockets and a higher probability of having more severe stages of periodontitis compared to non-Sámi, when controlling for age, sex, education, smoking habits and dental attendance. In general, half of the participants with Sámi affiliation had periodontitis, and two out of ten had a stage III/IV periodontitis, i.e. severe periodontitis. Prevalence and severity increased with age and lower education level. Men had more severe periodontitis compared with women, and smokers had more periodontitis than non-smokers.

Comparing the prevalence of periodontitis in this study with previous findings in Norway is not straightforward because different case definitions have been used. The prevalence of periodontitis in the current study was in range of the results from another study on adults in Troms County, northern Norway [14, 25] where almost half of the study population had periodontitis and about 9% had severe periodontitis when classified according to the CDC/AAP definition [26]. Holde [25] re-classified the same study population using the new case definition according to AAP/EFP [23, 24, 27], and found that the total prevalence of periodontitis (Stage I-IV) was 48%. Prevalence of Stage II was 19.5, and 20.8% of the participants were classified as having severe periodontitis i.e. Stage III/IV. The findings are consistent with findings in the current study regarding Stage III/IV. However, the prevalence of stage II was around 50% higher in the present study, which means that the total prevalence of periodontitis was higher in Finnmark. The prevalence of periodontitis in adults differs between European countries, and has been reported to be around 50% in Germany [28], 33–40% in Sweden [18, 19, 29], and 64% Finland [20, 30]. However, because different case definitions for periodontitis were used, any direct comparisons should be made with caution.

Pocket depths ≥4 mm or ≥ 6 mm (one or more pockets), the basic clinical measurement for the preliminary diagnosis of periodontitis, is often reported. In the present study the prevalence of PD ≥4 mm and PD ≥ 6 mm was higher among the Sámi participants compared with non-Sámi, but the the proportion of individuals with PD ≥6 mm in the present study was in range with findings from previous studies of comparable age groups in northern Norway (18.7%) [14], and in Oslo (8%) [15]. However, in the oldest age group (65–75 year) the proportions of periodontitis in the present study were somewhat lower compared to a study of Norwegian pensioners (33%) [16]. Comparing the present findings with other Scandinavian studies, the prevalence of individuals with periodontal pockets (≥ 4 mm) was lower than in a Swedish study (75%) [19], but in range with a Finnish study (64%) [20]. Edman et al. [29] used the alveolar bone loss (ABL) in premolars and molars to investigate and classify the severity and prevalence of periodontitis in an adult population (20–85 years) in Sweden, and found that 33% of the study population had moderate bone loss and 6% had severe bone loss. Comparing this findings with the present study, the prevalence of moderate bone loss was equal, but severe bone loss was more common in northern Norway than in study from Sweden.

Smoking is a factor strongly associated with periodontal disease [31]. Consistent with previous studies [32, 33] the odds of having periodontitis was higher among smokers than non- smokers. The number of smokers in the present study was higher than among adults in Troms County (15%) [14], and also higher than the national averages in 2013 (15%) [34], which could have had an impact on the higher prevalence of total periodontitis. Furthermore, Finnmark County has a history of irregular access to dentists, dental hygienists and/or specialists [35], and the distance to a dental clinic could have made it difficult for inhabitants to seek treatment. Studies have shown that higher availability of dentists decreased the likelihood of periodontitis [31, 36].

Previous studies have shown differences in periodontal health between indigenous and non-indigenous people around the world [5, 12]. In the present study the prevalence of periodontitis was not higher in the indigenous Sámi population compared to non-Sámi. However, there were significantly younger individuals in the Sámi group and when controlling for age, the Sámi had higher odds of having periodontitis.

More persons with Sámi affiliation did not brush their teeth daily. A nearly 30-year old study from Finnmark also reported that participants with Sámi affiliation had no regular tooth brushing habits in their childhood, which affected the tooth brushing habits as adults [2] This can be seen in the context of the Sámi culture, where the Sámi people raise their children with more freedom and less regular routines [37].

The main strength of the present study is the large sample size (*n* = 2078), the high response rate (88.7%) and the large number of participants with Sámi affiliation (*n* = 1381). As large parts of the traditional Sámi settlement regions were included, the findings of prevalence of periodontitis in Sámi people could be regarded representative of the Sámi population living in northern Norway. This study has methodological limitations, as described in Brustad et al. [22], where the external validity of the study was questioned because participants in the study were patients at the public dental care services, and not randomly chosen from the population. This may have affected the results and estimates and thus may not represent the situation for the whole population in this region. However, in the area where this study was conducted very few private dentists are available. Thus, most of the inhabitants seeking dental care go to a Public Dental clinic (as paying patients) in the area where they live. There are also limitations due to the clinical dental examination and the post clinical measurements of bone level. The alveolar bone level was measured on premolars and molars on bitewing, and RBL was estimated from mean length on premolars and molars. Not knowing the exact length of the root could mostly affect the measurement of RBL < 15%, and because of that, Stage I periodontitis was classified as non-severe periodontitis. This could lead to an underestimation of early stages of periodontitis in the population, but may not affect the classification of more severe periodontitis, as stage II and stage III. On the other hand, this study had a full-mouth examination protocol on PD. Brustad et al. [22] concluded that the validity of measurements used in the presents study was acceptable, which is a strength of the study.

## Conclusion

The prevalence of periodontitis was high in communities in the core area of Sámi settlement in Northern Norway, regardless of ethnicity. People with Sámi ethnicity had more deep periodontal pockets and an increased likelihood of having severe stages of periodontitis. Future studies should address possible explaining factors behind the potential higher risk of having more severe periodontitis among indigenous people in Sámi settlements.

## Data Availability

The datasets generated and/or analyzed during the current study are not publicly available due EU GPDR regulation, we cannot make the data into so called “open access data”.

## Abbreviations

AAP: American Academy of Periodontology
ABL: Alveolar bone level
A-KSB: Ann-Kristine Sara Bongo
aOR: Adjusted odds ratio
CAL: Clinical attachment level
EFP: European Federation of Periodontology
NSP: non-severe periodontitis
NO: Nils Oscarson
OR: Odds ratio
PD: Periodontal probing depth
RBL: Radiographic bone loss
SD: Standard deviation

## Footnotes

1 AAP/EFP classification is based on a World workshop on the classification of Periodontal and Peri-Implant Diseases co-sponsored by the American Academy of Periodontology (AAP) and European Federation of Periodontology (EFP)
