# Correction to: Periodontal health in an indigenous Sámi population in Northern Norway: a cross-sectional study

**DOI:** 10.1186/s12903-021-01631-y

**Published:** 2021-07-07

**Authors:** Ann-Kristine Sara Bongo, Magritt Brustad, Nils Oscarson, Birgitta Jönsson

**Affiliations:** 1 Department of Community Medicine, Faculty of Health Sciences, UiT the Arctic University of Norway, Tromsø, Norway; 2 The Public Dental Health Service Competence Centre of Northern Norway (TkNN), P.O Box 2406, N-9271 Tromsø, Norway; 3 Sámi University of Applied Science, Kautokeino, Norway; 4 Department of Periodontology, Institute of Odontology, The Sahlgrenska Academy, University of Gothenburg, Gothenburg, Sweden

## Correction to: BMC Oral Health (2020) 20:104 https://doi.org/10.1186/s12903-020-01098-3

Following publication of the original article [1], the authors identified an error in the Table 4.

The correct table is given below:

**Table 4:** Prevalence and extent of radiographic bone loss and periodontal pocked depth by age group, ethnicity and in total

Periodontal measurements	Age Groups (years)								Total	
	18–34		35–49		50–64		65–75		18–75	
Ethnicity	Sámi	Non-Sámi	Sámi	Non-Sámi	Sámi	Non-Sámi	Sámi	Non-Sámi	Sámi	Non-Sámi
Number of individuals	313	106	435	252	468	241	165	98	1381	697
RBL, proportions^a^										
RBL 15–33%	3.8	5.7	**32.6**	**25.0**	59.4	53.5	53.9	57.1	37.7	36.4
RBL > 33%	0.3	0.0	3.5	5.6	23.3	21.2	25.5	25.5	12.1	12.9
PD, proportions^a^										
One or more PD ≥ 4 mm	40.6	40.6	63.7	56.4	**71.6**	**63.1**	73.3	81.6	62.3	59.8
One or more PD ≥ 6 mm	5.1	5.7	**16.1**	**10.3**	**29.1**	**19.9**	27.3	27.5	**19.4**	**15.6**
PD, mean (SE)^b^										
Proportions of sites/mouth										
PD ≥ 4 mm	3.5 (0.4)	3.8 (0.7)	7.7 (0.5)	6.8 (0.7)	10.5 (0.7)	10.1 (1.0)	8.8 (0.9)	11.7 (1.4)	8.0 (0.3)	8.2 (0.5)
PD ≥ 6 mm	0.2 ( 0.0)	0.1 (0.1)	0.5 (0.1)	0.4 (0.1)	1.3 (0.2)	1.1 (0.2)	1.2 (0.2)	1.2 (0.3)	0.8 (0.1)	0.7 (0.1)

RBL = radiographic bone loss; PD = periodontal pocket depth. SE = standard error. Bold-face = statistically significant differences between Sámi and non-Sámi in each age group (*p* < 0.05). Statistical analyses were done with ^a^ = χ^2^-test and ^b^ = independent t-test

Furthermore, due to those errors in Table 4, following corrections are related to the text:

1. Statistical analysis on page 3, paragraph 4: ‘RBL and PD are presented as percent and proportions (SE) of affected sites and teeth for the total study population, stratified by age group and ethnicity. Differences between groups were assessed with χ^2^-test and t-test;
2. Results on page 4 under subtitle ‘Prevalence and distribution’, last sentence: ‘In total (18–75 years), a higher proportion of Sámi had one or more PD ≥ 6 mm compared to non-Sámi (*p* < 0.05.);
3. Discussion on page 5, third paragraph, second sentence: ‘A larger proportion of Sámi had one or more PD ≥ 6 mm compared to non-Sámi, with no ethnic difference in prevalence of PD ≥ 4 mm and PD ≥ 6 mm’;
4. Abstract on page 1 under results, last sentence: ‘A higher proportion of Sámi had one or more PD ≥ 6 mm than the non-Sámi (*p* < 0.05)’;

Abstract on page 1 under conclusion: ‘People with Sámi ethnicity had deep periodontal pockets and an increased odds of having severe stages of periodontitis.

The original article has been updated.
